# Clinical outcomes in older adults with advanced thyroid cancer

**DOI:** 10.1093/oncolo/oyag200

**Published:** 2026-05-20

**Authors:** Helena J Janse van Rensburg, Tian Xiao, Katherine Lajkosz, Vijithan Sugumar, Shabbir M H Alibhai, David Goldstein, Aruz Mesci, Carly C Barron, Lucy X Ma

**Affiliations:** Division of Medical Oncology and Hematology, Princess Margaret Cancer Centre, University Health Network, University of Toronto, Toronto, ON M5G 2M9, Canada; Division of Medical Oncology and Hematology, Princess Margaret Cancer Centre, University Health Network, University of Toronto, Toronto, ON M5G 2M9, Canada; Division of Medical Oncology and Hematology, Princess Margaret Cancer Centre, University Health Network, University of Toronto, Toronto, ON M5G 2M9, Canada; Division of Medical Oncology and Hematology, Princess Margaret Cancer Centre, University Health Network, University of Toronto, Toronto, ON M5G 2M9, Canada; Department of Supportive Care, Princess Margaret Cancer Centre, University Health Network, University of Toronto, Toronto, ON M5G 2M9, Canada; Department of Otolaryngology—Head and Neck Surgery, Princess Margaret Cancer Centre, University Health Network, University of Toronto, Toronto, ON M5G 2M9, Canada; Department of Radiation Oncology, Princess Margaret Cancer Centre, University Health Network, University of Toronto, Toronto, ON M5G 2M9, Canada; Division of Medical Oncology and Hematology, Princess Margaret Cancer Centre, University Health Network, University of Toronto, Toronto, ON M5G 2M9, Canada; Division of Medical Oncology and Hematology, Princess Margaret Cancer Centre, University Health Network, University of Toronto, Toronto, ON M5G 2M9, Canada

**Keywords:** thyroid neoplasms, geriatric oncology, systemic therapy, targeted therapy, tyrosine kinase inhibitors

## Abstract

**Background:**

Age is a prognostic factor in thyroid cancer. However, little is known about benefits and risks of systemic therapy in older adults with advanced thyroid cancer.

**Patients and methods:**

We performed a retrospective review of patients ≥ 65 years of age with advanced thyroid cancer referred to medical oncology at our institution for consideration of systemic therapy between 2010 and 2025 (*n *= 114). Clinicopathologic, treatment, adverse event, and outcomes data were analyzed.

**Results:**

65/114 patients received first-line systemic therapy. Median overall survival (OS) was 4.04 years (95% CI, 3.09-not estimable) in the whole cohort and 3.91 years (95% CI, 2.84-not estimable) in the treated cohort. First-line time-to-treatment discontinuation (TTD) in the treated cohort was 1.59 years (95% CI, 0.75-4.45). Age, male sex, and anaplastic histology predicted shorter OS in the whole cohort. Age, male sex, increasing Charlson comorbidity index, and *BRAF* and/or *TERT* mutation predicted worse OS and/or TTD amongst treated patients. For patients receiving lenvatinib (*n *= 37), adverse events led to significant proportions of patients requiring dose reductions (65%), treatment breaks (78%), emergency department (ED) visits (16%), and drug discontinuation (14%). Adverse events led to treatment breaks (56%), ED visits (67%), and drug discontinuation (22%) in patients receiving dabrafenib + trametinib (*n *= 9). Female sex, geriatric oncology referral, polypharmacy, and *BRAF* mutation predicted higher number of ED visits.

**Conclusion:**

Although survival outcomes were comparable to those described in younger adults, adverse events were common and impactful in older adults.

Implications for Practice Older adults with cancer have unique considerations for cancer diagnosis, prognosis, and management. There is limited clinical trial evidence studying the efficacy and toxicities of systemic therapies in older adults with advanced thyroid cancer such that oncologists must depend on expert guidance and individual experience to inform treatment recommendations. The data presented in the current study addresses this knowledge gap and may be useful in guiding discussions around the benefits and risks of systemic therapy in this population.

## Introduction

Older adults represent a special and heterogeneous population in oncology.^1^ When discussing palliative-intent systemic therapy with older adult patients, oncologists are challenged with the task of contextualizing potential benefits of treatment amongst competing risk from other life-limiting diagnoses and overall life expectancy. At the same time, clinicians must consider the impact of possible adverse effects of therapy on quality of life directly or through the exacerbation of comorbid conditions where these exist. For patients on treatment requiring dose adjustment or interruption, there must be continual reflection upon whether efficacy is maintained to justify ongoing use and associated risks. With limited clinical trial evidence specifically testing individual therapies in older adults, data to inform such decisions can be lacking leading oncologists to rely upon expert guidance and personal experience for treatment recommendations.

Advanced age has been identified as a prognostic factor in thyroid cancer patients. Indeed, thyroid cancers arising in older adults have more aggressive disease biology, with high risk histologic (eg, poorly differentiated, anaplastic) and genomic (eg, *BRAF*, *TERT* mutations) features being more frequently observed.^2–5^ Age has been uniquely incorporated into the AJCC staging system for thyroid cancer primarily due to favourable outcomes in younger patients.^6^ In the setting of advanced disease, age can markedly impact therapeutic decisions. Older adults have been found to have less radioactive iodine (RAI)-avid disease and issues with safe administration in renal, hepatic, or cardiac impairment can limit its utility.^7^^,^^8^ Although locoregional therapies may be preferred for slow-growing or low-volume disease, older adults may not be felt to be suitable candidates.^9^ As a result, more patients may be deemed RAI-resistant and/or unresectable prompting referral to medical oncology for consideration of systemic therapy.^10^

We have recently reported treatment outcomes in patients receiving first-line lenvatinib for advanced follicular cell-derived thyroid cancer at our institution.^11^ In a univariable analysis, increasing age was associated with shorter time-to-treatment discontinuation (TTD) and was further found to be a key variable in distinguishing patients who were “excellent responders” from those that were “poor responders.” Despite these preliminary observations, little is known about the benefits and risks of systemic therapy in older adults with advanced thyroid cancer and there are no national or international guidelines addressing this issue.

In the present study, we describe our single-center experience with a cohort of older adults with advanced thyroid cancer referred to medical oncology for consideration of systemic therapy. We summarize first-line systemic therapy decisions and provide survival outcomes for the whole and treated cohorts. Baseline clinicopathologic features associated with worse survival outcomes are highlighted. Finally, adverse effects of interest as well as clinicopathologic predictors of these are explored in patients receiving lenvatinib or dabrafenib + trametinib. We expect that these findings will be useful in informing discussions surrounding targeted therapy for older adult patients with advanced thyroid cancer.

## Patients and methods

### Study design and patient cohort

The endocrine oncology program at the University Health Network—Princess Margaret Cancer Centre maintains a database of patients with advanced thyroid cancer (defined as unresectable localized, unresectable locally advanced, recurrent, or distant metastatic disease) referred to medical oncology for consideration of systemic therapy. Only patients with RAI-resistant disease (defined as non-avid disease, cumulative lifetime dose ≥600 mCi, or progression within 12 months of RAI) or contraindications to RAI are considered for systemic therapy. We performed a retrospective chart review of patients who were ≥ 65 years of age at the time of consultation from 2010 to 2025 (*n *= 114). Electronic medical records were reviewed for demographics, medical history, disease characteristics, treatment details, adverse events, and clinical outcomes (data cut-off: August 6, 2025). Next-generation sequencing was performed on tumour samples through the Integrated Molecular Profiling in Advanced Cancers (IMPACT; NCT1505400) or Ontario-wide Cancer TArgeted Nucleic Acid Evaluation (OCTANE; NCT02906943) clinical trials or through the Access to Genetic Advanced Testing (AGATE) institutional initiative. Informed consent for participation in these programs was obtained and methods have been described.^12^^,^^13^ This study was approved by the University Health Network Research Ethics Board (CAPCR 24-6124).

### Statistical analysis

Demographic and clinical data were analyzed using descriptive statistics. Data are presented as means, medians, and ranges or as absolute numbers of patients and proportions where specified. TTD and overall survival (OS) were analyzed using Kaplan–Meier methods. TTD was defined as the time from systemic therapy initiation until discontinuation for any reason. OS was determined from the date of medical oncology consultation for analyses involving the whole patient population and from the date of first-line systemic therapy start for analyses involving treated patients only. Patients were censored at their last contact date for time-to-event analyses. Univariable Cox proportional hazards models were used to test for associations between clinicopathological variables and TTD or OS. Subsequent multivariable analyses were conducted with variables from the univariable analyses (based on *P *< .15) incorporated into a stepwise regression algorithm. All features with *P *<.15 were retained in the final models. For adverse event analyses, the cumulative number of selected adverse events or emergency department (ED) visits were modelled as counts using Poisson regression models. Subsequent multivariable analyses were conducted as described. Statistical analyses were performed using R Statistical Software.^14^ Statistical significance was defined as *P *<.05.

## Results

### Cohort characteristics

A total of 114 patients ≥ 65 years of age were referred to medical oncology for consideration of systemic therapy between 2010 and 2025. Baseline characteristics are described in Table 1 while prior locoregional therapies are summarized in Table S1. In brief, the median age of patients was 73 (range 65-98) with most being between 65 and 74. The majority of patients spoke English as their first language (74%), lived with at least one other person (72%), were functionally independent (93%), and had an ECOG performance status of 0 or 1 (88%).

**Table 1.** Demographic and clinical characteristics of patients at baseline (*n *= 114).

Demographics		Medical history		Primary disease	
**Age**		**Charlson Comorbidity Index** ^a^		**Age at initial diagnosis**	
** Median, years (range)**	73 (65-98)	**0**	70 (61%)	**Median, years (range)**	68 (21-86)
** Distribution, # (%)**		**1**	18 (16%)	**Distribution, # (%)**	
** 65-74**	73 (64%)	**2**	15 (13%)	**≤ 64**	47 (41%)
** 75-84**	36 (32%)	**3**	8 (7%)	**65-74**	54 (47%)
** ≥ 85**	5 (4%)	**4+**	3 (3%)	**≥ 75**	13 (11%)
**Sex, # (%)**		**Comorbid mood disorder, # (%)**	15 (13%)	**Predominant histology, # (%)**	
** Male**	65 (57%)	**Comorbid cognitive disorder** ^b^ **, # (%)**	0 (0%)	**Papillary**	67 (59%)
** Female**	49 (43%)	**Prior malignancy, # (%)**	26 (23%)	**Follicular**	3 (3%)
**First language, # (%)**		**Skin**	10 (38%)	** Oncocytic**	3 (3%)
** English**	85 (74%)	**Breast**	6 (23%)	**Poorly differentiated**	20 (18%)
** Non-English**	29 (25%)	**Renal**	5 (19%)	** Anaplastic**	17 (15%)
**Living situation, # (%)**		**Prostate**	3 (12%)	**Multiple/other**	4 (4%)
** Alone**	23 (20%)	**Bladder**	2 (8%)	**Sites of disease # (%)**	
** With ≥ 1 other person**	82 (72%)	**Hepatopancreatobiliary**	2 (8%)	**Local/locoregional disease**	49 (43%)
** Unknown**	9 (8%)	**Endometrial**	1 (4%)	**Local/locoregional only**	11 (10%)
**Functional status, # (%)**		**Lung**	1 (4%)	**Distant**	
** Independent ADLs/IADLs**	106 (93%)	**Lymphoma**	1 (4%)	**Non-regional LN**	31 (27%)
** Dependent IADLs only**	7 (6%)	**Other**	1 (4%)	**Bone**	28 (24%)
** Dependent ADLs/IADLs**	1 (1%)	**Multiple prior malignancies, # (%)**	6 (5%)	**Lung**	91 (80%)
**Smoking history, # (%)**		**Polypharmacy** ^c^ **, # (%)**	68 (60%)	**Pleura/pleural effusion**	6 (5%)
** Never**	65 (57%)	**BMI # (%)**		**Liver**	7 (6%)
** Current**	4 (4%)	** Underweight (<18.5)**	1 (1%)	**Brain**	13 (11%)
** Prior**	43 (38%)	**Healthy (18.5-24.9)**	30 (26%)	**Other**	11 (10%)
** Unknown**	2 (2%)	**Overweight (25-29.9)**	40 (35%)	**NGS**	
**Alcohol history, # (%)**		**Obese (30-39.9)**	30 (26%)	**Alteration in… # (%)**	
** Never**	45 (39%)	**Severely obese (≥40)**	3 (3%)	* **BRAF***	52 (46%)
** Current occasional**	46 (40%)	**Unknown**	10 (9%)	* **TERT***	36 (32%)
** Current daily**	12 (10%)			* **TP53***	14 (12%)
** Prior**	6 (5%)			*** NRAS***	13 (11%)
** Unknown**	5 (4%)			* **HRAS***	6 (5%)
**Performance status, # (%)**				**≥ 2 alterations**	64 (56%)
** ECOG0**	39 (34%)			**Cumulative RAI dose (mCi), # (%)**	
** ECOG1**	62 (54%)			**None**	20 (18%)
** ECOG2**	6 (5%)			**100-299**	55 (48%)
** ECOG3**	1 (1%)			**300-599**	26 (23%)
** ECOG4**	0 (0%)			**≥ 600**	2 (2%)
** Unknown**	6 (5%)			**Unknown**	11 (10%)

Abbreviations: ADLs, activities of daily living; BMI, Body mass index; ECOG, Eastern Cooperative Oncology Group; IADLs, instrumental activities of daily living; LN, lymph node; NGS, next-generation sequencing; RAI, radioactive iodine.

a Not including points for age and thyroid cancer diagnosis.

b Including dementia, mild cognitive impairment, and cognitive impairment not otherwise specified.

c Defined as ≥5 prescription medications.

The most common Charlson Comorbidity Index (CCI) score (not including points for age and thyroid cancer diagnosis) amongst the patients was 0. Comorbid mood and cognitive disorders were infrequent at 13% and 0%, respectively. In contrast, polypharmacy (defined as taking at least five prescription medications) was observed in 60% of patients. A prior diagnosis of malignancy was reported by 23% of patients and 5% of patients carried a history of more than one prior malignancy.

Papillary thyroid cancer was the most common thyroid cancer histology (59%), whereas fewer patients had follicular (3%), anaplastic (15%), or poorly differentiated (18%) disease. 90% of patients had distant metastases with lung (80%), bone (24%), and non-regional nodal (27%) disease being the most frequent sites of involvement. NGS testing revealed BRAF-V600E mutations in 46% of patients while 32% of patients had *TERT* promoter mutations. Finally, most patients had prior exposure to RAI (73%).

### Determination of treatment plan and selection of first-line therapy

**Figure 1.**
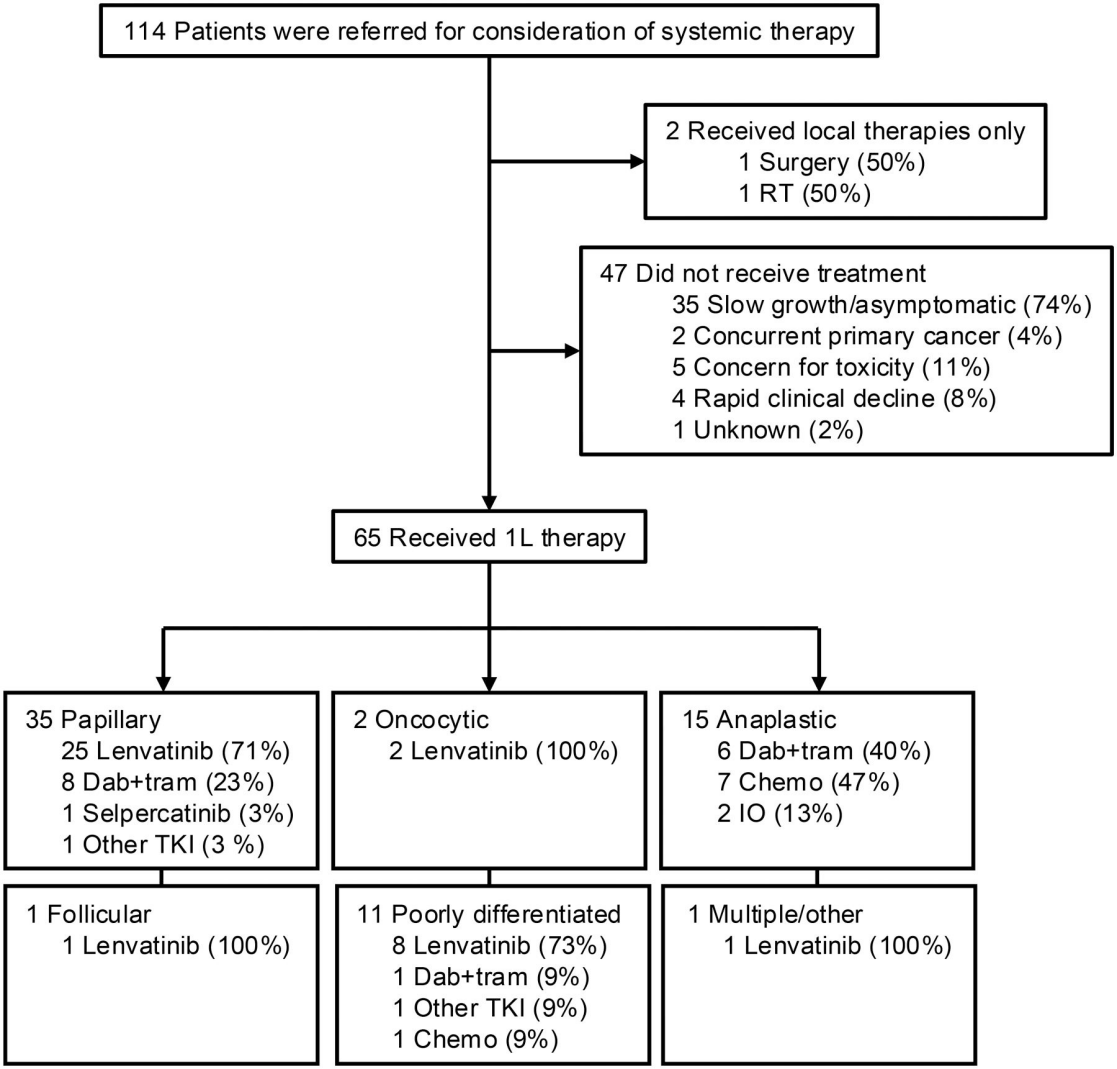
Diagram of first-line systemic therapy selection for older adults with advanced thyroid cancer. 1 L, first-line; Dab+tram, dabrafenib + trametinib; IO, immunotherapy; RT, radiation therapy; TKI, tyrosine kinase inhibitor.

Out of 114 patients referred to medical oncology, 47 patients (41%) did not receive any therapy (Figure 1). Local modalities (ie, surgery or radiation) were preferred for two patients. Systemic therapy was not felt to be indicated due to slow growing or asymptomatic disease in a majority of those who did not receive treatment (74%). Two patients (4%) had treatment deferred due to a concurrent primary cancer and five others (11%) did not receive treatment due to concerns for toxicity. Four patients (8%) experienced a rapid clinical decline that precluded treatment. Amongst the 65 patients (57%) who did receive treatment, a variety of agents were prescribed with treatment choice influenced by tumour histology, anticipated side effects, contraindications to specific therapies, and results of NGS. 37 patients were prescribed lenvatinib. Dabrafenib + trametinib was the second most common regimen and was prescribed to 15 patients.

### Involvement of geriatric oncology

Nine patients in the cohort were referred to our geriatric oncology clinic (Table S2). The most common reason for referral was concerns for cognition (55%). Three patients were referred for pre-treatment assessment and others were referred for concerns related to mobility or mood. A variety of interventions were performed by the geriatricians in this clinic including referrals to allied health (arranged for 56% of patients), specialty MDs, and home-visiting MDs. Practitioners further arranged for supportive equipment, adjusted supportive medications, de-prescribed medications, ordered bloodwork or imaging investigations, and arranged for community supports. Two patients had a recommendation made for tyrosine kinase inhibitor (TKI) dose adjustment. Most patients referred to geriatric oncology (78%) did proceed to receive systemic therapy.

### Survival outcomes in the whole and treated cohorts

**Figure 2.**
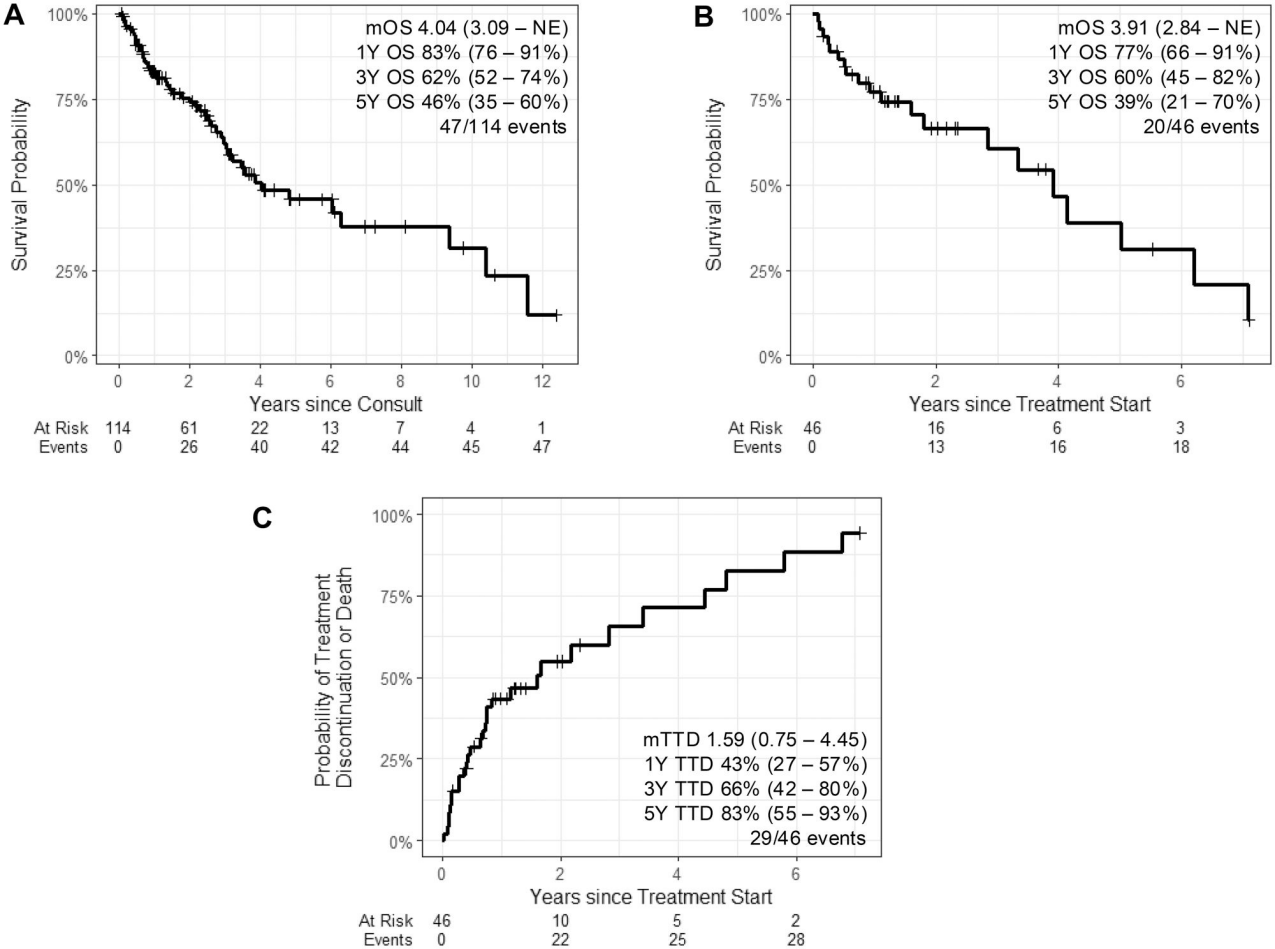
A. Overall survival in whole cohort (treated and untreated) of older adults with advanced thyroid cancer. B. Overall survival and time-to-treatment discontinuation (C) in geriatric patients with advanced non-anaplastic thyroid cancer treated with first-line lenvatinib or dabrafenib + trametinib. Median overall survival (mOS) and time-to-treatment discontinuation (mTTD), as well as 1-year (1Y OS/TTD), 3-year (3Y OS/TTD), and 5-year (5Y OS/TTD) values shown in insets. 95% CI depicted in parentheses. NE, non-estimable.

Patients in the whole cohort were followed for a median of 26.5 months (range 0.9-148 months) from initial consultation. The median overall survival (mOS) was 4.04 years (95% CI, 3.09 years-not estimable) (Figure 2A). 83% (95% CI, 76-91) of patients were alive at one year after consultation, 62% (95% CI, 52-74) of patients were alive at three years, and 46% (95% CI, 35-60) of patients were alive at five years. OS by histology is shown in Figure S1. The median follow-up time from initial consultation was 29.9 months (range 1.7-138 months) in those patients with non-anaplastic histology who received treatment with lenvatinib or dabrafenib + trametinib (“treated cohort”, baseline characteristics in Table S3) and the median follow-up time from treatment initiation in these patients was 15.4 months (range 1.1-85 months). The mOS for this cohort was 3.91 years (95% CI, 2.84 years-not estimable) with 77% (95% CI, 66-91) of patients alive at one year after treatment start, 60% (95% CI, 45-82) alive at three years, and 39% (95% CI, 21-70) alive at five years (Figure 2B). The median time-to-treatment discontinuation (mTTD) in the treated cohort was 1.59 years (95% CI, 0.75-4.45 years) with 43% (95% CI, 27-57), 66% (95% CI, 42-80), and 83% (95% CI, 55-93) of patients having discontinued treatment or died at one, three, and five years after treatment start, respectively (Figure 2C).

### Factors associated with survival outcomes

Multivariable analyses were performed to identify patient characteristics associated with survival outcomes in the whole and treated cohorts (Table 2). Age (HR 1.09 per year, 95% CI, 1.03-1.15), male sex (HR 1.99, 95% CI, 1.06-3.71), and anaplastic histology (HR 4.63, 95% CI, 2.23-9.62) were significantly associated with shorter OS in the whole study population. In the treated cohort, age (HR 1.09 per year, 95% CI, 1.00-1.17), higher CCI (HR 1.52 per point, 95% CI, 1.01-2.30), and presence of a *BRAF* and *TERT* co-mutation (HR 7.24, 95% CI, 1.66-31.53) were associated with shorter OS. Similarly, male sex (HR 3.37, 95% CI, 1.27-8.93) and presence of a *BRAF* mutation (HR 4.57, 95% CI, 1.49-14.07), *TERT* mutation (HR 4.49, 95% CI, 1.07-18.92), or *BRAF* and *TERT* co-mutation (HR 4.39, 95% CI, 1.49-12.93) were associated with shorter TTD in the treated cohort. Conversely, polypharmacy conferred a longer TTD in the treated cohort (HR 0.41, 95% CI, 0.18-0.94).

**Table 2:** Multivariable analyses for factors associated with survival outcomes.

OS in whole cohort	HR (95% CI)	*P*-value
**Age, per year**	1.09 (1.03-1.15)	.004
**Sex**		
** Female**	Reference	
** Male**	1.99 (1.06-3.71)	.032
**Predominant histology**		
** Well differentiated**	Reference	
** Poorly differentiated/oncocytic (*n* = 23)**	2.02 (0.96-4.26)	.063
** Anaplastic (*n* = 17)**	4.63 (2.23-9.62)	< .001
**Local disease**		
** No**	Reference	
** Yes**	1.71 (0.92-3.15)	.088

**OS in treated cohort**	**HR (95% CI)**	***P*-value**

**Age, per year**	1.09 (1.00-1.17)	.042
**Charlson Comorbidity Index, per point**	1.52 (1.01-2.30)	.046
***BRAF/TERT* status**		
** Both wild-type**	Reference	
** *BRAF*-mutated only**	2.73 (0.67-11.17)	.16
** *TERT*-mutated only**	4.09 (0.76-22.08)	.10
** *BRAF-* and *TERT*-mutated**	7.24 (1.66-31.53)	.008

**TTD in treated cohort**	**HR (95% CI)**	***P*-value**

**Sex**		
** Female**	Reference	
** Male**	3.37 (1.27-8.93)	.015
**Charlson Comorbidity Index, per point**	1.41 (0.98-2.04)	.066
***BRAF/TERT* status**		
** Both wild-type**	Reference	
** *BRAF*-mutated only**	4.57 (1.49-14.07)	.008
** *TERT*-mutated only**	4.49 (1.07-18.92)	.041
** *BRAF-* and *TERT*-mutated**	4.39 (1.49-12.93)	.007
**Polypharmacy^a^**		
** No**	Reference	
** Yes**	0.41 (0.18-0.94)	.035

Abbreviations: OS, overall survival; TTD, time-to-treatment discontinuation.

a Defined as ≥5 prescription medications.

### Dosing and adverse events of lenvatinib therapy

A total of 37 patients were prescribed lenvatinib (Table 3). The most common starting dose was 10 mg daily. 65% of patients required at least one dose reduction during treatment. The most common maintenance dose was 4 mg daily (range 4-14 mg). Adverse events were common with ≥ 5% body weight loss (73%), hypertension requiring a medication change (57%), diarrhea (38%), and hand-foot syndrome (HFS, 27%) being frequently reported. 78% of patients required at least one treatment break due to toxicities. 54% of all treatment breaks were related to adverse events of lenvatinib whereas 24% were related to medical interventions (ie, procedures, hospital admissions, etc.) and the remainder were for other causes including patient preference. Five patients (14%) discontinued lenvatinib due to toxicities. 59% of patients had at least one ED visit while on lenvatinib and 16% of patients had at least one ED visit that was related to lenvatinib.

**Table 3:** Dose adjustments and adverse events of lenvatinib (*n *= 37).

Dose		Discontinuation	
**Most common starting dose, mg (range)**	10 (4-24)	**Patients with discontinuation due to AE, # (%)**	5 (14%)
**Average starting dose, mg**	12.9	**Discontinuation due to …, # (% of discontinuation due to AE)**	
**Patients with ≥ 1 dose reduction, # (%)**	24 (65%)	**Proteinuria**	1 (20%)
**Most common maintenance dose, mg (range)**	4 (4-14)	**Skin wound**	1 (20%)
**Average maintenance dose, mg**	8.5	**Weakness/fatigue**	2 (40%)
		**Hemolytic anemia**	1 (20%)
**Selected adverse events**			
**Patients experiencing …, # (%)**		**ED visits**	
**Hypertension requiring medication change**	21 (57%)	**Patients with ≥ 1 ED visit, # (%)**	22 (59%)
**Diarrhea**	14 (38%)	**Total number ED visits**	53
**≥ 5% weight loss**	27 (73%)	**Average # ED visit/patient with ED visit (range)**	2.4 (1-8)
**HFS**	10 (27%)	**Patients with ≥ 1 ED visit tx-related, # (%)**	6 (16%)
**Mucositis**	8 (22%)	**Average # ED visit tx-related/patient with ED visit tx-related (range)**	1.3 (1-2)
**Proteinuria**	8 (22%)	**ED visit due to… , # (% of ED visits)**	
		**Primary diagnosis**	10 (19%)
**Other adverse events**		**Tumour symptom**	3 (6%)
**Patients experiencing …, # (%)**		**Complication of cancer (eg, VTE, pain, bleeding)**	6 (11%)
**Electrolyte abnormalities^a^**	9 (24%)	**Medical device issue**	1 (2%)
**Hyperglycemia^b^**	3 (8%)	**Tx-related**	8 (15%)
		**Hypertension**	4 (8%)
**Treatment breaks**		**Diarrhea**	1 (2%)
**Patients with ≥ 1 treatment break, # (%)**	29 (78%)	**HFS**	2 (4%)
**Total number treatment breaks**	123	**Hemolytic anemia**	1 (2%)
**Average # treatment break/patient with treatment break (range)**	4.2 (1-18)	**Other**	35 (66%)
**Patients with ≥ 1 treatment break AE-related, # (%)**	29 (78%)	**Confusion**	2 (4%)
**Average # tx break AE-related/patient with tx break AE-related (range)**	2.3 (1-11)	** Weakness/fatigue**	3 (6%)
**Treatment break due to… , # (% of treatment breaks)**		**Infection**	5 (9%)
**AE-related**	67 (54%)	**Comorbidity**	5 (9%)
**Hypertension**	11 (9%)	**Trauma**	4 (8%)
**Diarrhea**	15 (12%)	**Unclassified (eg, other symptom, lab abnormality)**	16 (30%)
**≥ 5% weight loss**	13 (10%)		
**HFS**	8 (6%)		
**Mucositis**	2 (2%)		
**Proteinuria**	2 (2%)		
**Low appetite**	3 (2%)		
**Skin wound**	7 (6%)		
**Weakness/fatigue**	6 (5%)		
**Intervention-related**	30 (24%)		
**Procedure (**eg**, dental, surgical)**	13 (10%)		
**Radiation therapy**	9 (7%)		
**Hospital admission**	7 (6%)		
**Drug-drug interaction**	1 (1%)		
**Other**	26 (21%)		
**Infection**	4 (3%)		
**Unclassified (**eg**, other symptom, lab abnormality)**	15 (12%)		
**Patient preference (**eg**, vacation)**	7 (6%)		

Abbreviations: AE, adverse events; ED, emergency department; HFS, hand-foot syndrome; Tx, treatment; VTE, venous thromboembolism.

a Defined as sodium <130 mmol/L, potassium <3 mmol/L, corrected calcium <2 mmol/L, or magnesium <0.5 mmol/L.

b Defined as fasting plasma glucose >7 mmol/L or random plasma glucose ≥11.1 mmol/L.

### Dosing and adverse events of dabrafenib + trametinib therapy

Nine patients with non-anaplastic thyroid cancer received dabrafenib + trametinib (Table S4). The most common starting dose was 150 mg BID + 2 mg daily. Two patients (22%) required at least one dose reduction. Fever, ≥ 5% body weight loss, hypertension requiring a medication change, and HFS were commonly described. 56% of patients required at least one treatment break due to toxicities from therapy. 81% of all treatment breaks were due to toxicities (69% specifically due to fever), and the remainder were due to medical interventions. Two patients discontinued dabrafenib + trametinib due to adverse events (both due to fever). 78% of patients had at least one ED visit during treatment, and 67% had at least one ED visit due to toxicities, with fever being responsible for 60% of total ED visits and all treatment-related ED visits. Additional causes for ED visits are outlined in Table S5.

### Factors associated with adverse event outcomes in treated patients

Exploratory multivariable analyses were performed to assess for characteristics associated with adverse events (Table 4). A higher cumulative number of selected adverse events was observed in patients who were referred to geriatric oncology (RR 1.93, 95% CI, 1.15-3.08). Polypharmacy (RR 2.17, 95% CI, 1.25-4.01), presence of a *BRAF* mutation (RR 2.28, 95% CI, 1.29-4.10), and geriatric oncology consultation (RR 2.51, 95% CI, 1.34-4.53) were associated with higher number of ED visits, whereas male sex was associated with fewer visits (RR 0.56, 95% CI, 0.34-0.91). Overall, there was no difference in the cumulative number of selected adverse events or ED visits between patients receiving lenvatinib or dabrafenib + trametinib (Table S6). However, there was an increased risk of treatment-related ED visits in patients receiving dabrafenib + trametinib as compared to lenvatinib. A trend towards increased cumulative number of ED visits was observed in patients with a *BRAF* mutation who received dabrafenib + trametinib as opposed to lenvatinib.

**Table 4:** Multivariable analyses for factors associated with adverse event outcomes.

Cumulative number of selected adverse events	RR (95% CI)	*P*-value
**Age, per year**	0.96 (0.92-1.00)	.051
**Sex**		
** Female**	Reference	
** Male**	0.71 (0.48-1.06)	.093
**Geriatric oncology consulted**		
** No**	Reference	
** Yes (*n* = 9)**	1.93 (1.15-3.08)	.009

**Number of ED visits**	**RR (95% CI)**	***P*-value**

**Sex**		
** Female**	Reference	
** Male**	0.56 (0.34-0.91)	.021
**Polypharmacy^a^**		
** No**	Reference	
** Yes**	2.17 (1.25-4.01)	.009
***BRAF/TERT* status**		
** Both wild-type**	Reference	
** *BRAF*-mutated only**	2.28 (1.29-4.10)	.005
** *TERT*-mutated only**	1.26 (0.51-2.83)	.59
** *BRAF-* and *TERT*-mutated**	1.27 (0.64-2.48)	.48
**Geriatric oncology consulted**		
** No**	Reference	
** Yes (*n* = 9)**	2.51 (1.34-4.53)	.003

Abbreviation: ED, emergency department.

a Defined as ≥ 5 prescription medications.

### Discontinuation of first-line therapy

Twenty-four of the 37 patients who received lenvatinib discontinued it during the follow-up period, with 50% of discontinuations being due to progression, 21% due to toxicity, 17% due to comorbidity, and 8% due to death (Table 5). Eight patients received second-line systemic therapy. Five of nine patients who received dabrafenib + trametinib discontinued it during the follow-up period (one due to progression, two due to toxicity, and two due to death). Two patients received second-line systemic therapy.

**Table 5:** First-line systemic therapy discontinuation amongst non-anaplastic patients.

Lenvatinib (*n* = 37)		Dabrafenib + Trametinib (*n* = 9)	
**Reason for discontinuation (*n* = 24)**		**Reason for discontinuation (*n* = 5)**	
** Progression**	12 (50%)	**Progression**	1 (20%)
** Toxicity**	5 (21%)	**Toxicity**	2 (40%)
** Comorbidity**	4 (17%)	**Death**	2 (40%)
** Death**	2 (8%)	**Received second-line therapy**	
** Unknown**	1 (4%)	**Yes**	2 (40%)
**Received second-line therapy**		**No**	3 (60%)
** Yes**	8 (33%)		
** No**	15 (62%)		
** Unknown**	1 (4%)		

## Discussion

Older adults with thyroid cancer have a higher risk of aggressive disease.^2–4^ Despite this, there is a lack of published data interrogating clinical outcomes of systemic therapy for older adults with advanced thyroid cancer. In the present study, we address this by describing our single-center experience with older adults with advanced thyroid cancer referred to medical oncology for consideration of first-line systemic therapy.

Most patients in our study proceeded to receive systemic therapy. Only a small number of patients did not receive treatment due to age-related reasons namely concerns for increased toxicity or competing risk from comorbidities. In practice, those who did not receive treatment were generally patients in whom treatment was not yet felt to be indicated due to asymptomatic, low volume, or indolent disease. Treatment decisions in our cohort were therefore generally reflective of those involving younger adults with advanced thyroid cancer as outlined in published guidelines.^15^

Although our whole and treated cohorts were comprised of patients with varying thyroid cancer histology and first-line therapies, survival outcomes were within the range of expected values from previous studies in younger-aged patients. In the SELECT trial (median patient age 64) as well as in a subsequent sub-analysis of SELECT with patients stratified by age, a similar proportion of patients were alive at one or two years after treatment start as observed in our treated cohort although mOS was not reached.^16^^,^^17^ In our prior study of first-line lenvatinib for patients with advanced follicular cell derived-thyroid cancer, the median age at treatment start was 62.9 years and mOS was 72.9 months, with modestly higher fractions of patients alive at one, three, and five years after treatment start as compared to our treated cohort.^11^ Survival outcomes were also in keeping with data from a phase II trial of dabrafenib + trametinib in patients with BRAF-V600E-mutated differentiated thyroid cancer with a median age of 65.^18^ Importantly, patients with anaplastic thyroid cancer were included in our whole cohort, impacting calculated survival outcomes. Nevertheless, our data shows that older adults with advanced thyroid cancer can experience prolonged survival warranting consideration of palliative-intent systemic therapy.

Our multivariable analyses identified factors associated with shorter OS and TTD. In the whole cohort, age and male sex were associated with higher risk of mortality. These variables both also appeared in a published nomogram predicting mortality in older adults with papillary thyroid cancer.^19^ Intuitively, increasing comorbidity burden and the presence of *BRAF* and/or *TERT* mutations were associated with shorter OS and/or TTD in the treated cohort. Both of these characteristics have been associated with worse prognosis.^20–22^ Polypharmacy was associated with longer TTD in the treated cohort. This was unexpected as polypharmacy has almost ubiquitously been identified as harmful in older adults with cancer.^23^ A rationale for this is unclear but may relate to polypharmacy as a surrogate marker for engagement with medical care and well-controlled comorbidities, including those with the potential to be exacerbated by targeted therapies (eg, hypertension, chronic kidney disease). As our analysis did not evaluate polypharmacy in depth (ie, with consideration for drug classes, potentially inappropriate medications, drug-drug interactions), this finding should be interpreted with caution.

A small sample of patients in our cohort were assessed by geriatric oncology, with abnormal cognition being the most common reason for referral. A particular emphasis upon understanding patients’ cognitive function may be warranted given that TKIs are self-administered at home and require some amount of independent monitoring (eg, blood pressure checks). This finding might also be reflective of underdiagnosis of cognitive impairment in our cohort with no patients carrying a formal diagnosis of such at initial consultation. Fewer patients were referred for a pre-treatment assessment. Certainly, whereas cytotoxic chemotherapy might trigger a clinician to consider geriatric assessment to evaluate for potential significant toxicities, oral targeted therapies may not prompt clinicians in the same way due to lower perceived risks. Indeed, commonly utilized toxicity calculators used for this purpose (eg, CARG and others) have not been validated with targeted therapies and may not be entirely applicable to rarer tumour sites.^24^ Regardless of the reason for referral, patients in our cohort benefitted from multiple interventions from geriatric oncology in keeping with guidelines from ASCO.^25^ Involvement of allied health was especially frequent, and this may be an intervention treating oncologists could readily apply when access to a formal geriatric oncology program is lacking.

Adverse events observed in our study were frequent and impactful, often requiring permanent dose modification, treatment interruption, ED visits, and/or drug discontinuation. Lenvatinib was routinely started at a dose lower than what has been recommended in guidelines from the American Thyroid Association (10 mg as compared to 24 mg).^15^ Despite this, most patients in our cohort required further dose reduction. There were no patients who received full dose lenvatinib as maintenance, and there were also no patients who received a maintenance dose corresponding to the median dose intensity of 16 mg that was previously described from the older adult SELECT sub-analysis.^17^ Nonetheless, our observation that survival outcomes were comparable to those previously published supports utilization of modified dosing in older adults.

As expected, hypertension was a common toxicity resulting in medication changes for more than half of patients, as well as treatment breaks and ED visits in a small proportion of patients. Some of the oncologists in our institution elected to prescribe antihypertensive medications preemptively when starting lenvatinib, although this should be considered on a case-by-case basis. Indeed, optimal blood pressure targets are the subject of debate even for healthy older adults.^26^ Additional reflection must be given towards potential risks of stringent blood pressure management in an older adult population, including electrolyte disturbances and falls.

Other toxicities of lenvatinib were in keeping with its known side effect profile. Weight loss was common but generally manageable through treatment breaks. Diarrhea, mucositis, and HFS were reported slightly less frequently than in the literature for younger patients. Delayed healing of skin wounds was an unexpected notable toxicity from our data, perhaps due to age-related changes in skin with increased vulnerability to minor trauma. As a result of these and other toxicities, a significant majority of patients in our treated cohort required at least one break from lenvatinib, while a non-negligible proportion of patients visited the ED because of treatment and/or had to discontinue the drug due to adverse events of therapy. Interestingly, the frequencies of dose interruption and discontinuation in our treated cohort were similar to those described in SELECT and the older adult sub-analysis.

A small sample of patients in our treated cohort received dabrafenib + trametinib, which was routinely started and maintained at full dose. Fever was the cause of most treatment breaks, ED visits, and toxicity-related drug discontinuation for these patients. Despite being impactful, fever was reported in the same proportion of our patients as described in younger adults.^18^ Although variable strategies were utilized for management in our cohort, consensus guidelines on the management of pyrexia with dabrafenib + trametinib in melanoma patients may provide insights into how this toxicity may be addressed while limiting treatment interruptions.^27^

We further performed multivariable analyses to investigate risk factors for adverse events of therapy. As expected, patients seen by geriatric oncology or with polypharmacy had a higher risk of cumulative selected adverse events with therapy and/or ED visits, probably due to selection bias towards a more vulnerable or comorbid population. Male sex was protective against ED visits which has been well-described.^28^ Interestingly, the presence of a *BRAF*-mutation was associated with a higher cumulative number of ED visits. This finding may be explained only in part due to the use of dabrafenib + trametinib in some of these patients as dabrafenib + trametinib was associated with increasing number of treatment-related ED visits (but not ED visits overall) in our treated cohort, and there was only a non-significant trend towards increasing number of ED visits with dabrafenib + trametinib use in *BRAF*-mutated patients.

To the best of our knowledge, this study offers the first real-world description of clinical outcomes for older adults with advanced thyroid cancer referred for potential systemic therapy. Our study benefits from a moderate sample size, lengthy follow-up period, and capture of multiple clinically relevant outcomes. However, several limitations should be considered. Our population included many younger, well-supported, and independent patients with few comorbidities. Whether our results are generalizable to an older, more vulnerable, comorbid, and/or frail population is unknown. The retrospective study design precluded grading of adverse events and also prevented us from being able to report measures with particular importance in the geriatric oncology population including physical fitness and frailty assessments, life expectancy, as well as toxicity scores.^29–31^ Although TSH suppression is an essential aspect of advanced thyroid cancer management, we were further unable to collect data pertaining to TSH suppression and levothyroxine dosing as this frequently occurred outside of our institution. Our adverse event multivariable analyses are limited by a small number of events and should be considered exploratory. Finally, given the retrospective study design with adverse event data collected from clinical notes as well as the fact that prior research has suggested that older adults may under-report treatment toxicities, the true burden of adverse events may be greater than what was described in our study.^32^ Nevertheless, our data provides insights into the benefits and risks of systemic therapy and should prove valuable for clinicians treating advanced thyroid cancer in framing treatment recommendations.

## Conclusion

In our study of older adults with advanced thyroid cancer, first-line treatment decisions were comparable to those made in younger adults. Survival outcomes were in the range of observed values from prior literature. However, adverse events of treatment were frequent, impactful, and must be weighed against expected benefits of treatment guided by patient values and preferences. Starting dose optimization and close monitoring based on an individual’s risk factors for adverse events should be considered.

## Supplementary Material

oyag200_Supplementary_Data

## Data Availability

The data underlying this article will be shared on reasonable request to the corresponding author.

## References

[1] Marosi C , Köller M. Challenge of cancer in the elderly. ESMO Open. 2016;1:e000020. 10.1136/esmoopen-2015-000020 27843603 PMC5070391

[2] Kwong N , Medici M, Angell TE, et al The influence of patient age on thyroid nodule formation, multinodularity, and thyroid cancer risk. J Clin Endocrinol Metab. 2015;100:4434-4440. 10.1210/jc.2015-3100 26465395 PMC4667162

[3] Papaleontiou M , Haymart MR. Approach to and treatment of thyroid disorders in the elderly. Med Clin North Am. 2012;96:297-310. 10.1016/j.mcna.2012.01.013 22443977 PMC3314224

[4] Shen X , Zhu G, Liu R, et al Patient age–associated mortality risk is differentiated by BRAF V600E status in papillary thyroid cancer. J Clin Oncol. 2018;36:438-445. 10.1200/JCO.2017.74.5497 29240540 PMC5807010

[5] Qasem E , Murugan AK, Al-Hindi H, et al TERT promoter mutations in thyroid cancer: a report from a Middle Eastern population. Endocr Relat Cancer. 2015;22:901-908. 10.1530/ERC-15-0396 26354077

[6] Amin MB , Greene FL, Edge SB, et al The eighth edition AJCC cancer staging manual: Continuing to build a bridge from a population-based to a more “personalized” approach to cancer staging. CA Cancer J Clin. 2017;67:93-99. 10.3322/caac.21388 28094848

[7] Tuttle RM , Leboeuf R, Robbins RJ, et al Empiric radioactive iodine dosing regimens frequently exceed maximum tolerated activity levels in elderly patients with thyroid cancer. J Nucl Med. 2006;47:1587-1591. 17015892

[8] Nakanishi K , Kikumori T, Miyajima N, et al Impact of patient age and histological type on radioactive iodine avidity of recurrent lesions of differentiated thyroid carcinoma. Clin Nucl Med. 2018;43:482-485. 10.1097/RLU.0000000000002078 29688947

[9] Joseph KR , Edirimanne S, Eslick GD. Thyroidectomy for thyroid cancer in the elderly: a meta-analysis. Eur J Surg Oncol. 2019;45:310-317. 10.1016/j.ejso.2018.07.055 30642604

[10] Agosto Salgado S , Kaye ER, Sargi Z, Chung CH, Papaleontiou M. Management of advanced thyroid cancer: Overview, advances, and opportunities. Am Soc Clin Oncol Educ Book. 2023;43:e389708. 10.1200/EDBK_389708 37186883

[11] Ruicci KM , Deng Y, Xiao T, et al Real-world treatment outcomes and clinicopathologic and molecular determinants of response to first-line lenvatinib in patients with advanced follicular cell-derived thyroid cancer. BJC Rep. 2025;3:41. 10.1038/s44276-025-00153-2 40461800 PMC12134128

[12] Stockley TL , Oza AM, Berman HK, et al Molecular profiling of advanced solid tumors and patient outcomes with genotype-matched clinical trials: the princess margaret IMPACT/COMPACT trial. Genome Med. 2016;8:109. 10.1186/s13073-016-0364-2 27782854 PMC5078968

[13] Malone ER , Saleh RR, Yu C, et al OCTANE (Ontario-wide cancer targeted nucleic acid evaluation): a platform for intraprovincial, national, and international clinical data-sharing. Curr Oncol. 2019;26:e618-e623. 10.3747/co.26.5235 31708655 PMC6821116

[14] RDevelopment Core Team. R: A Language and Environment for Statistical Com- Puting. 4.4.1 ed. Vienna, Austria: R Foundation for Statistical Computing; 2010.

[15] Ringel MD , Sosa JA, Baloch Z, et al 2025 American thyroid association management guidelines for adult patients with differentiated thyroid cancer. Thyroid. 2025;35:841-985. 10.1177/10507256251363120 40844370 PMC13090833

[16] Schlumberger M , Tahara M, Wirth LJ, et al Lenvatinib versus placebo in Radioiodine-Refractory thyroid cancer. N Engl J Med. 2015;372:621-630. 10.1056/NEJMoa1406470 25671254

[17] Brose MS , Worden FP, Newbold KL, Guo M, Hurria A. Effect of age on the efficacy and safety of lenvatinib in Radioiodine-Refractory differentiated thyroid cancer in the phase III SELECT trial. J Clin Oncol. 2017;35:2692-2699. 10.1200/JCO.2016.71.6472 28613956

[18] Busaidy NL , Konda B, Wei L, et al Dabrafenib versus dabrafenib + trametinib in BRAF-Mutated radioactive iodine refractory differentiated thyroid cancer: Results of a randomized, phase 2, Open-Label multicenter trial. Thyroid. 2022;32:1184-1192. 10.1089/thy.2022.0115 35658604 PMC9595631

[19] Wang J , Zhanghuang C, Jin L, et al Development and validation of a nomogram to predict cancer-specific survival in elderly patients with papillary thyroid carcinoma: a population-based study. BMC Geriatr. 2022;22:736. 10.1186/s12877-022-03430-8 36076163 PMC9454205

[20] Xing M , Westra WH, Tufano RP, et al BRAF mutation predicts a poorer clinical prognosis for papillary thyroid cancer. J Clin Endocrinol Metab. 2005;90:6373-6379. 10.1210/jc.2005-0987 16174717

[21] Lee YK , Hong N, Park SH, et al The relationship of comorbidities to mortality and cause of death in patients with differentiated thyroid carcinoma. Sci Rep. 2019;9:11435. 10.1038/s41598-019-47898-8 31391492 PMC6685995

[22] Liu R , Xing M. TERT promoter mutations in thyroid cancer. Endocr Relat Cancer. 2016;23:R143-R155. 10.1530/ERC-15-0533 26733501 PMC4750651

[23] Balducci L , Goetz-Parten D, Steinman MA. Polypharmacy and the management of the older cancer patient. Ann Oncol. 2013;24 Suppl 7:vii36-vii40. 10.1093/annonc/mdt266 24001761 PMC6278993

[24] Johnson WV , Gupta A, Blaes AH, et al Oncologist perspectives on the acceptability, appropriateness, and feasibility of the cancer and aging research group (CARG) chemotherapy toxicity prediction tool for older adults. J Geriatr Oncol. 2025;16:102303. 10.1016/j.jgo.2025.102303 40580678

[25] Dale W , Klepin HD, Williams GR, et al Practical assessment and management of vulnerabilities in older patients receiving systemic cancer therapy: ASCO guideline update. J Clin Oncol. 2023;41:4293-4312. 10.1200/JCO.23.00933 37459573 PMC12803700

[26] Jamshidian MS , Scherzer R, Estrella MM, et al Individualized net benefit of intensive blood pressure lowering among Community-Dwelling older adults in SPRINT. J Am Geriatr Soc. 2025;73:1441-1453. 10.1111/jgs.19395 39967308 PMC12100678

[27] Thawer A , Miller WH, Gregorio N, et al Management of pyrexia associated with the combination of dabrafenib and trametinib: Canadian consensus statements. Curr Oncol. 2021;28:3537-3553. 10.3390/curroncol28050304 34590600 PMC8482100

[28] Aalam AA , Iftikhar N, Baskaran N, Bhat A. Exploring gender disparities in emergency department utilization: a comprehensive comparative analysis of the frequency of female versus male emergency department visits. Cureus. 2024;16:e68066. 10.7759/cureus.68066 39347320 PMC11438533

[29] Schonberg MA , Davis RB, McCarthy EP, Marcantonio ER. Index to predict 5-Year mortality of Community-Dwelling adults aged 65 and older using data from the national health interview survey. J Gen Intern Med. 2009;24:1115-1122. 10.1007/s11606-009-1073-y 19649678 PMC2762505

[30] Hurria A , Togawa K, Mohile SG, et al Predicting chemotherapy toxicity in older adults with cancer: a prospective multicenter study. J Clin Oncol. 2011;29:3457-3465. 10.1200/JCO.2011.34.7625 21810685 PMC3624700

[31] Rockwood K , Song X, MacKnight C, et al A global clinical measure of fitness and frailty in elderly people. CMAJ. 2005;173:489-495. 10.1503/cmaj.050051 16129869 PMC1188185

[32] Cataldo JK , Paul S, Cooper B, et al Differences in the symptom experience of older versus younger oncology outpatients: a cross-sectional study. BMC Cancer. 2013;13:6. 10.1186/1471-2407-13-6 23281602 PMC3576303

