# Supplementary material for: Clinical outcomes in older adults with advanced thyroid cancer
Source: Oncologist. 2026 May 20;31(7):oyag200. doi: 10.1093/oncolo/oyag200 (PMC13261072; doi:10.1093/oncolo/oyag200)
Supplement: oyag200_Supplementary_Data [file oyag200_supplementary_data.zip › 4. Supplementary Tables 1-6.docx]

| **Supplementary Table 1. Surgical, radiation, and radioactive iodine history of patients at baseline (*n* = 114)** | | | | | | |  |  |
| --- | --- | --- | --- | --- | --- | --- | --- | --- |
| SURGERY |  |  | RADIATION |  |  | RADIOACTIVE IODINE |  | |
| Primary surgery |  |  | Total courses |  |  | Total courses |  | |
| Partial thyroidectomy | 9 (8%) |  | Median (range) | 1 (0 - 10) |  | 0 | 20 (18%) | |
| Total thyroidectomy | 87 (76%) |  | 0 | 35 (31%) |  | 1 | 34 (30%) | |
| Unknown | 18 (16%) |  | 1 - 2 | 55 (48%) |  | 2 | 43 (38%) | |
| Second surgery |  |  | 3 - 4 | 13 (11%) |  | ≥ 3 | 16 (14%) | |
| Locoregional disease | 44 (39%) |  | ≥ 5 | 8 (7%) |  | Unknown | 1 (1%) | |
| Distant metastatic disease | 14 (12%) |  | Unknown | 3 (3%) |  | Indication |  | |
| None | 56 (49%) |  | Indication |  |  | Adjuvant only | 25 (22%) | |
| Third surgery |  |  | Adjuvant | 21 (18%) |  | Metastatic disease only | 19 (17%) | |
| Locoregional disease | 16 (14%) |  | Metastatic disease | 41 (36%) |  | Both | 46 (40%) | |
| Distant metastatic disease | 10 (9%) |  | Unknown | 17 (15%) |  | None | 20 (18%) | |
| None | 88 (77%) |  | None | 35 (31%) |  | Unknown | 4 (4%) | |

| **Supplementary Table 2. Geriatric oncology consults and recommendations (*n* = 9)** | |
| --- | --- |
| REFERRAL |  |
| Single reason for referral, # (%) | 7 (78%) |
| ≥ 2 reasons for referral, # (%) | 2 (22%) |
| Reason for referral … |  |
| Pre-treatment assessment | 3 (27%) |
| Mobility | 1 (9%) |
| Mood | 1 (9%) |
| Cognition | 6 (55%) |
|  |  |
| RECOMMENDATIONS…, # (%) | |
| TKI dose adjustment | 2 (22%) |
| Depression medication adjustment | 1 (11%) |
| Cognition medication adjustment | 1 (11%) |
| Supportive medication adjustment^a^ | 2 (22%) |
| Medication de-prescribed | 2 (22%) |
| Ordered investigations | 2 (22%) |
| Allied health referral | 5 (56%) |
| Specialty MD referral | 3 (33%) |
| Home-visiting MD referral | 2 (22%) |
| Supportive equipment provided | 3 (33%) |
| Community supports provided | 2 (22%) |
| Home care arranged | 1 (11%) |
| POA documentation | 1 (11%) |
|  |  |
| FOLLOW-UP |  |
| Appointment provided, # (%) | 6 (67%) |
|  |  |
| TREATMENT |  |
| First-line therapy received, # (%) | 7 (78%) |

^a^Includes analgesia, bowel regimen, etc. TKI: tyrosine kinase inhibitor. POA: power of attorney.

| **Supplementary Table 3. Demographic and clinical characteristics of patients with non-anaplastic thyroid cancer treated with lenvatinib or dabrafenib + trametinib at baseline (*n* =46)** | | | | | | | |
| --- | --- | --- | --- | --- | --- | --- | --- |
| DEMOGRAPHICS |  |  | MEDICAL HISTORY |  |  | PRIMARY DISEASE |  |
| Age |  |  | Charlson Comorbidity Index^a^ |  |  | Age at initial diagnosis |  |
| Median, years (range) | 73 (65 - 86) |  | 0 | 26 (57%) |  | Median, years (range) | 66 (27 - 80) |
| Distribution, # (%) |  |  | 1 | 7 (15%) |  | Distribution, # (%) |  |
| 65 – 74 | 32 (70%) |  | 2 | 8 (17%) |  | ≤ 64 | 21 (46%) |
| 75 – 84 | 11 (24%) |  | 3 | 4 (9%) |  | 65 – 74 | 20 (43%) |
| ≥ 85 | 3 (6%) |  | 4+ | 1 (2%) |  | ≥ 75 | 5 (11%) |
| Sex, # (%) |  |  | Comorbid mood disorder, # (%) | 4 (9%) |  | Predominant histology, # (%) |  |
| Male | 28 (61%) |  | Comorbid cognitive disorder^b^, # (%) | 0 (0%) |  | Papillary | 33 (72%) |
| Female | 18 (39%) |  | Prior malignancy, # (%) | 12 (26%) |  | Follicular | 1 (2%) |
| First language, # (%) |  |  | Skin | 8 (67%) |  | Oncocytic | 2 (4%) |
| English | 33 (72%) |  | Breast | 2 (17%) |  | Poorly differentiated | 9 (20%) |
| Non-English | 13 (28%) |  | Renal | 2 (17%) |  | Anaplastic | 0 (0%) |
| Living situation, # (%) |  |  | Prostate | 2 (17%) |  | Multiple/other | 1 (2%) |
| Alone | 12 (26%) |  | Bladder | 1 (8%) |  | Sites of disease # (%) |  |
| With ≥ 1 other person | 32 (70%) |  | Multiple prior malignancies, # (%) | 3 (7%) |  | Local/locoregional disease | 13 (28%) |
| Unknown | 2 (4%) |  | Polypharmacy^c^, # (%) | 30 (65%) |  | Local/locoregional only | 2 (4%) |
| Functional status, # (%) |  |  | BMI # (%) |  |  | Distant |  |
| Independent ADLs/IADLs | 43 (93%) |  | Underweight (< 18.5) | 0 (0%) |  | Non-regional LN | 15 (33%) |
| Dependent IADLs only | 3 (6%) |  | Healthy (18.5 – 24.9) | 13 (28%) |  | Bone | 17 (37%) |
| Dependent ADLs/IADLs | 0 (0%) |  | Overweight (25 – 29.9) | 15 (33%) |  | Lung | 39 (85%) |
| Smoking history, # (%) |  |  | Obese (30 – 39.9) | 15 (33%) |  | Pleura/pleural effusion | 4 (9%) |
| Never | 25 (54%) |  | Severely obese (≥ 40) | 1 (2%) |  | Liver | 3 (7%) |
| Current | 2 (4%) |  | Unknown | 2 (4%) |  | Brain | 9 (20%) |
| Prior | 19 (41%) |  |  |  |  | Other | 6 (13%) |
| Alcohol history, # (%) |  |  |  |  |  | NGS |  |
| Never | 21 (46%) |  |  |  |  | Alteration in… # (%) |  |
| Current occasional | 19 (41%) |  |  |  |  | *BRAF* | 23 (50%) |
| Current daily | 4 (9%) |  |  |  |  | *TERT* | 16 (35%) |
| Prior | 2 (4%) |  |  |  |  | *TP53* | 5 (11%) |
| Performance status, # (%) |  |  |  |  |  | *NRAS* | 4 (9%) |
| ECOG0 | 16 (35%) |  |  |  |  | *HRAS* | 2 (4%) |
| ECOG1 | 28 (61%) |  |  |  |  | ≥ 2 alterations | 26 (57 %) |
| ECOG2 | 0 (0%) |  |  |  |  | Cumulative RAI dose (mCi), # (%) |  |
| ECOG3 | 1 (2%) |  |  |  |  | None | 8 (17%) |
| ECOG4 | 0 (0%) |  |  |  |  | 100 – 299 | 22 (48%) |
| Unknown | 1 (2%) |  |  |  |  | 300 – 599 | 14 (30%) |
|  |  |  |  |  |  | ≥ 600 | 2 (4%) |

^a^Not including points for age and thyroid cancer diagnosis. ^b^Including dementia, mild cognitive impairment, and cognitive impairment not otherwise specified. ^c^Defined as ≥ 5 prescription medications. ADLs: activities of daily living. BMI: Body mass index. ECOG: Eastern Cooperative Oncology Group. IADLs: instrumental activities of daily living. LN: lymph node. NGS: next-generation sequencing. RAI: radioactive iodine.

| **Supplementary Table 4. Dose adjustments and adverse events of dabrafenib + trametinib (*n* = 9)** | | | |  | |  | |
| --- | --- | --- | --- | --- | --- | --- | --- |
| **Dabrafenib + trametinib (*n* = 9)** |  |  |  | |  | |  |
| DOSE |  |  | DISCONTINUATION | |  | |  |
| Most common starting dose, mg^a^ | 150 BID/2 |  | Patients with discontinuation due to AE, # (%) | | 2 (22%) | |  |
| Average starting dose, mg^a^ | 133 BID/2 |  | Discontinuation due to …, # (% of discontinuation due to AE) | |  | |  |
| Patients with ≥ 1 dose reduction, # (%) | 2 (22%) |  | Fever | | 2 (100%) | |  |
| Most common maintenance dose dabrafenib, mg (range) | 150 (75 - 150) |  |  | |  | |  |
| Most common maintenance dose trametinib, mg (range) | 2 (1.5 - 2) |  |  | |  | |  |
| Average maintenance dose, mg^a^ | 128 BID/2 |  |  | |  | |  |
|  |  |  |  | |  | |  |
| SELECTED ADVERSE EVENTS |  |  | ED VISITS | |  | |  |
| Patients experiencing …, # (%) |  |  | Patients with ≥ 1 ED visit, # (%) | | 7 (78%) | |  |
| Hypertension requiring medication change | 2 (22%) |  | Total number ED visits | | 20 | |  |
| Diarrhea | 1 (11%) |  | Average # ED visit/patient with ED visit (range) | | 2.9 (1 - 5) | |  |
| ≥ 5% weight loss | 4 (44%) |  | Patients with ≥ 1 ED visit tx-related, # (%) | | 6 (67%) | |  |
| HFS | 2 (22%) |  | Average # ED visit tx-related/patient with ED visit tx-related (range) | | 1.7 (1 - 4) | |  |
| Mucositis | 0 (0%) |  | ED visit due to… , # (% of ED visits) | |  | |  |
| Proteinuria | 0 (0%) |  | Primary diagnosis | | 3 (15%) | |  |
| Fever | 6 (67%) |  | Complication of cancer (e.g., VTE, pain, bleeding) | | 1 (5%) | |  |
|  |  |  | Medical device issue | | 2 (10%) | |  |
| OTHER ADVERSE EVENTS |  |  | Tx-related | | 12 (60%) | |  |
| Patients experiencing …, # (%) |  |  | Fever | | 12 (60%) | |  |
| Electrolyte abnormality^b^ | 1 (11%) |  | Other | | 5 (25%) | |  |
| Hyperglycemia^b^ | 1 (11%) |  | Confusion | | 1 (5%) | |  |
|  |  |  | Fall | | 2 (10%) | |  |
| TREATMENT BREAKS |  |  | Unclassified (e.g., other symptom, lab abnormality) | | 2 (10%) | |  |
| Patients with ≥ 1 treatment break, # (%) | 5 (56%) |  |  | |  | |  |
| Total number treatment breaks | 16 |  |  | |  | |  |
| Average # treatment break/patient with treatment break (range) | 3.2 (1 - 5) |  |  | |  | |  |
| Patients with ≥ 1 treatment break AE-related, # (%) | 5 (56%) |  |  | |  | |  |
| Average # tx break AE-related/patient with tx break AE-related (range) | 2.6 (1 - 4) |  |  | |  | |  |
| Treatment break due to… , # (% of treatment breaks) |  |  |  | |  | |  |
| AE-related | 13 (81%) |  |  | |  | |  |
| HFS | 1 (6%) |  |  | |  | |  |
| Weakness/fatigue | 1 (6%) |  |  | |  | |  |
| Fever | 11 (69%) |  |  | |  | |  |
| Intervention-related | 3 (19%) |  |  | |  | |  |
| Hospital admission | 3 (19%) |  |  | |  | |  |

^a^Depicted as dabrafenib dose (mg)/trametinib dose (mg). ^b^Defined as sodium < 130 mmol/L, potassium < 3 mmol/L, corrected calcium < 2 mmol/L, or magnesium < 0.5 mmol/L. ^c^Defined as fasting plasma glucose > 7 mmol/L or random plasma glucose ≥ 11.1 mmol/L. AE: adverse events. ED: emergency department. HFS: hand-foot syndrome. Tx: treatment. VTE: venous thromboembolism.

| **Supplementary Table 5. Expanded description of other causes for ED visits amongst treated patients** | | | | |  | |
| --- | --- | --- | --- | --- | --- | --- |
| **Lenvatinib (*n* = 37)** |  |  | **Dabrafenib + trametinib (*n* = 9)** |  | |  |
| ED visit due to… , # (% of ED visits) |  |  | ED visit due to… , # (% of ED visits) |  | |  |
| Other | 35 (66%) |  | Other | 5 (25%) | |  |
| Unclassified (e.g., other symptom, lab abnormality) | 16 (30%) |  | Unclassified (e.g., other symptom, lab abnormality) | 2 (10%) | |  |
| Abdominal pain | 2 (4%) |  | Dysphagia | 1 (5%) | |  |
| Chest pain | 2 (4%) |  | Hypotension | 1 (5%) | |  |
| Dental pain | 1 (2%) |  |  |  | |  |
| Groin pain | 1 (2%) |  |  |  | |  |
| Hemoptysis | 2 (4%) |  |  |  | |  |
| Hyponatremia | 2 (4%) |  |  |  | |  |
| Rash | 1 (2%) |  |  |  | |  |
| Shortness of breath | 4 (8%) |  |  |  | |  |
| Sore throat | 1 (2%) |  |  |  | |  |

ED: emergency department.

| **Supplementary Table 6. Comparison of adverse event outcomes by therapeutic agent** | | |  | |
| --- | --- | --- | --- | --- |
| WHOLE COHORT | RR (95% CI) | *p*-value | |  |
| Cumulative number of selected adverse events |  |  | |  |
| Lenvatinib | Reference |  | |  |
| Dabrafenib + trametinib | 0.71 (0.4 - 1.20) | 0.23 | |  |
| Number of ED visits |  |  | |  |
| Lenvatinib | Reference |  | |  |
| Dabrafenib + trametinib | 1.51 (0.88 - 2.48) | 0.12 | |  |
| Number of treatment-related ED visits |  |  | |  |
| Lenvatinib | Reference |  | |  |
| Dabrafenib + trametinib | 10 (2.07 - 59.46) | 0.006 | |  |
| *BRAF*-MUTATED PATIENTS | RR (95% CI) | *p*-value | |  |
| Cumulative number of selected adverse events |  |  | |  |
| Lenvatinib | Reference |  | |  |
| Dabrafenib + trametinib | 0.93 (0.47 - 1.73) | 0.82 | |  |
| Number of ED visits |  |  | |  |
| Lenvatinib | Reference |  | |  |
| Dabrafenib + trametinib | 1.81 (0.98 - 3.30) | 0.053 | |  |

ED: emergency department.
