# Supplementary material for: Clinical outcomes in older adults with advanced thyroid cancer
Source: Oncologist. 2026 May 20;31(7):oyag200. doi: 10.1093/oncolo/oyag200 (PMC13261072; doi:10.1093/oncolo/oyag200)
Supplement: oyag200_Supplementary_Data [file oyag200_supplementary_data.zip › 5. Supplementary Figure 1.pdf]

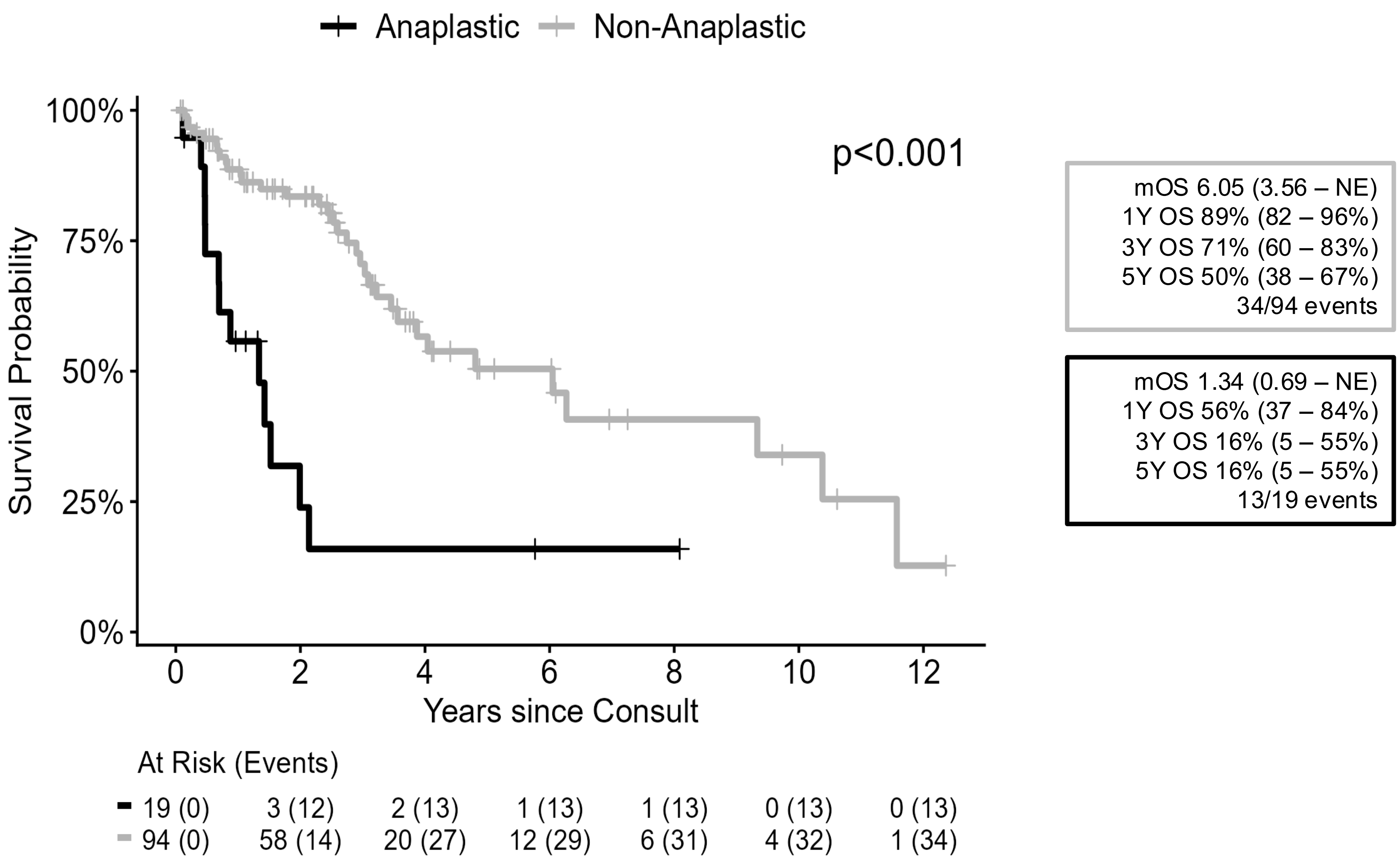

Supplementary Figure 1. Overall survival in whole cohort (treated and untreated) of older adults with advanced thyroid cancer by anaplastic (black line) and non-anaplastic (grey line) histology. Median overall survival (mOS) as well as 1-year (1Y OS), 3-year (3Y OS), and 5-year (5Y OS) values shown in insets (black for anaplastic, grey for non-anaplastic). 95% CI depicted in parentheses. NE: non-estimable.
